# Knowledge and beliefs on cervical cancer and practices on cervical cancer screening among women aged 20 to 50 years in Ouagadougou, Burkina Faso, 2012: a cross-sectional study

**DOI:** 10.11604/pamj.2014.18.175.3866

**Published:** 2014-06-23

**Authors:** Bernard Sawadogo, Sheba N Gitta, Elizeus Rutebemberwa, Mamadou Sawadogo, Nicola Meda

**Affiliations:** 1 Centre of International Research for Health, University of Ouagadougou, Burkina Faso; 2 African Field Epidemiology Network, Kampala, Uganda

**Keywords:** Cervical cancer, screening, knowledge, beliefs, practices

## Abstract

**Introduction:**

In Burkina Faso, 1230 women are diagnosed with cervical cancer every year and 838 die from the disease. Little is known about women's practices, knowledge and beliefs regarding cervical cancer. This study aims to describe women's practices regarding cervical cancer screening and to assess their knowledge and beliefs.

**Methods:**

Cross-sectional study was carried out in Ouagadougou from 1st to 31st December 2012 interviewing 840 women aged 20 to 50 years about their knowledge, beliefs and practices regarding cervical cancer. Cluster sampling was used. Univariate and multivariate logistic regression analysis were performed. Chi square test was used and p-value < 0.05 was considered.

**Results:**

Out of 840 women enrolled with mean age 29.5±7.77 years, 66.31% were married, 59.28% have not been to school or left school at primary level. While 64.2% of participants heart about cervical cancer, 8.5% heart about Human papillomavirus, 69.05% don't know that cervical cancer is preventable. 90.4% of participants were worried to develop cervical cancer, 96.67% would accept to be screened and 11.07% were screened for cervical cancer. In multivariate analysis, heart about cervical cancer (OR = 5.7; 95% CI: 2.21-14.69), know contamination mode of HPV (OR = 3.81; 95% CI: 2.27-6.39), heart about HPV (OR = 2.05; 95% CI: 1.11-3.81) and use of oral contraceptive (OR = 2.06; 95% CI: 1.25-3.39) were independently associated with screening history with p < 0.05.

**Conclusion:**

Knowledge and belief regarding cervical cancer is limited among Ouagadougou women and screening rate is low. There is need to enhance health education regarding Human papillomavirus and cervical cancer.

## Introduction

Cervical cancer is the third most commonly diagnosed cancer and the fourth leading cause of cancer death in female worldwide accounting for 9% (529 800) of the total new cancer cases and 8% (275100) of the total cancer deaths among females in 2008 [1]. Of these new cases of cervical cancer, 80% occur in developing countries [1, 2]. Cervical cancer has profound societal impact because it primarily affects women from their 30's to their 50's, who are often raising or supporting families. The burden of cervical cancer is potentially large in Sub-Saharan Africa and there is an urgency to make it a public health priority [3]. In west Africa, of total of 1 017 confirmed diagnosis of cancer, the three most common cancers were invasive cervical cancer (26.2%), breast cancer (23.1%) and non-Hodgkin's lymphoma (9.6%) [4]. Burkina Faso has a population of 3.8 million women ages 15 years and older who are at risk of developing cervical cancer and every year 1 230 women are diagnosed with cervical cancer and 838 die from the disease [5]. It is the most frequent cancer among women between 15 and 44 years of age [6, 7]. A case-control study conducted in Mali found that high parity, poor genital hygiene, increased number of sexual partner or common use of prostitute by husband are risk factors for cervical cancer and HPV DNA is present in almost all invasive cervical cancer [8]. Persistent human papillomavirus (HPV) infection is strongly linked to the development of cervical intraepithelial neoplasia grade 3 or carcinoma in situ “precancer” which can lead to cervical cancer [8, 9]. About 21.5% of women in the general population are estimated to harbour. cervical HPV infection at a given time, and 50% of invasive cervical cancers are attributed to HPV 16 or 18. Studies conducted in Burkina Faso reported HPV prevalence of 24.3% in women attending gynecology clinic [10], 59.6% in HIV-positive women [11] and 66.1% in female sex worker population [12]. HPV infection and development of cervical cancer can be prevented by health education, vaccination and early screening and treatment [13]. Cervical cancer screening is available in Ouagadougou but the screening coverage is low 5.5% [5]. Study conducted in East, Central and Southern Africa reported that reasons given by health workers why very few women are screened were the absence of policy guidelines, frequent shortage of materials needed taking Pap smear and long distance and cost of sending the smear to the processing centre [14]. However, it is possible to sustain good-quality visual screening service in low-income country [15] and lower average cost per women screened can be reach by screening high number of women [16]. Increasing knowledge of women on cervical cancer may increase screening coverage [17]. In Burkina Faso, the Papanicolaou (Pap) test, which is the most commonly performed test in developed countries, is limited to one national public hospital and two private hospitals in Ouagadougou. To response to this inaccessibility of the pap test by majority of women, JHPIEGO in collaboration with the Ministry of Health trained nurses and midwifes on alternative of cervical cancer screening know as visual inspection. Cervical cancer screening by visual inspection is available in some public and private hospital in Ouagadougou and in some provinces. Also mass campaign screening is organized in some provinces to offer opportunity of screening to rural women. Although the cervical cancer screening is available in Ouagadougou, the cervical cancer screening coverage is low. Little is known about Burkina Faso women's knowledge and beliefs about cervical cancer and screening. There is need to understand and address the multifaceted health beliefs that are likely to influence women's willingness to schedule and obtain screening. Assessing women knowledge on cervical cancer will help to increase perception of risk or change attitude toward cervical cancer screening and create demand for cervical cancer screening service. The purpose of this study, therefore, is to assess the knowledge and beliefs of women of Ouagadougou in order to provide health education area to enhance cervical cancer screening.

This study aims to describe the knowledge and beliefs on cervical cancer and practices on cervical cancer screening in women aged 20 to 50 years living in Ouagadougou and specifically to determine practices on cervical cancer in women aged 20-50 years in Ouagadougou and to assess Ouagadougou women’ knowledge, attitude and belief on cervical cancer.

## Methods

### Design, area and population

A cross-sectional study was carried out in Ouagadougou, capital of Burkina Faso. Ouagadougou is divided into 30 administrative sectors and 17 villages and 5 municipalities. It has 1.48 million inhabitants and 460 000 women aged 20 to 50 years in 2006. Most of the screening services are offered in Ouagadougou and women from other provinces are access to cervical screening service only during some mass campaign screening. The study population was women from 20 to 50 years living in Ouagadougou in 2012. Were included in this study, women aged 20-50 living in Ouagadougou for at least 6 months and had signed consent form. Were excluded women very ill, women mentally unstable and women currently enrolled on a clinical trial on cervical cancer.

### Sampling and sample size

We were assisted by one biostatician from Bernhard Nocht Institute for Tropical Medicine, Hamburg. Ouagadougou is divided into 30 administrative sectors. We used Google Earth software to plot randomly 30 points on the map of Ouagadougou corresponding to administrative sectors. For each administrative sector, we randomly chose 5 clusters and visited 5 household per sector. In each household we interviewed one eligible person. Sample size was 30 (sectors) x 5 (clusters) x 5 (households) = 750 persons. In prevision of non respondents, 830 participants were interviewed.

### Study variable

Dependent variables were practices on cervical cancer i.e. cervical cancer screening history with Pap test and visual inspection. Independent variables were sociodemographic characteristics (age, marital status, residence, occupation, income), risk behavior factors (smoking, oral contraceptive use, sexual debut, number of sexual partners, history of STI, parity), knowledge on cervical cancer (information on cervical cancer, diagnosis of cervical cancer, prevention of cervical cancer, information on HPV, transmission of HPV), and attitude (worry to develop cervical cancer, acceptance of cervical cancer screening).

### Data Collection

Interviewers were trained on administration of the questionnaire during two days. During the training, each interviewer and the supervisor applied each question on subject similar to those who were measured in the study. A simple listing of different answers gave an indication of whether these are due to carelessness, or show a consistent error. A pilot study was conducted in one sector to test the feasibility of the sampling, the data collection, the measurement methods and the questionnaire. Data collection was from 1st to 31st December 2012. Supervision reviewed every completed questionnaire, ensuring that all the questions have been asked and all the questionnaire instructions have been followed. Questionnaire that were incomplete or incorrectly completed were given back to the interviewer and the appropriate section re-administered. A telephone call also was made daily to know the number of questionnaire filled. All questions were checked as soon as possible after they were returned. Where information was missing or unclear, the interviewer was asked to resolve the query. This check provided particularly useful in filling gaps in the information and reconciling inconsistencies. In addition, it enabled the investigator to identify interviewers who were continuing to make some mistake and to provide those interviewers with necessary help or feedback to improve their work.

### Data management and analysis

Double entry was used to enter data in Epi Info version 3.5.1. This double entry helped to detect and correct errors and inconsistencies in the database not detected during the first quality check. Missing information was added by going back to the paper questionnaire. Data analysis was done using also Epi Info version 3.5.1. Univariate and multivariate logistic regression analysis were performed. The chi-square test was used and p-value < 0.05 was considered and excel software was used to draw graphs.

### Ethical consideration

Burkina Faso Ethics Committee for Research in Health approval was obtained and permission to carry out the study was obtained from Burkina Faso Ministry of Health. All respondents signed inform consent. The identity of respondents was not recorded on the questionnaire. The participants got information about cervical cancer.

## Results

Table 1 show that 840 respondents were enrolled. Majority of participants (64.60%) were young female less than 30 years with an average age of 29.37±7.77 years. Out of these 66.31% were married while 28.33% were single. Secondary or university education level participants were 40.3% and 41.3% of participants were housewife. Majority of participants (75.4%) have got their first sexual intercourse before 19 years and 45.6% of participants had history of sexually transmitted disease.

**Table 1:** Socio-demographic characteristics of participants on cervical cancer

Socio-demographic characteristics	Number (n = 840)	Percentage
**Age group**		
≤30 years	543	64.60
31-40 years	205	24.40
≥41 years	92	11
**Marital status**		
Married	557	66.31
Single	238	28.33
Divorced/Widowed	45	05.36
**Education level**		
Secondary/University	342	40.7
Not schooled	257	30.6
Primary	241	28.7
**Occupation**		
Housewife	347	41.3
Trader	188	22.4
Student	162	19.3
Employed	143	17
**Number of children**		
0	210	25
1-4	553	65.8
5-10	77	9.2
**Number of pregnancy**		
0	183	21.8
1-4	536	63.8
5-12	121	14.4
**Age first sex**		
≤19	633	75.4
≥20	207	24.6
**Number of sex partners**		
1	458	54.5
2-4	368	43.8
5 +	14	1.7
**History of STI**		
No	452	54.4
Yes	383	45.6
**Oral contraceptive use**		
Yes	383	45.6
No	457	54.4
**Family history of cervical cancer**		
No	820	97.62
Yes	20	02.38

Table 2 shows that out of 840, only 301 (35.8%) of participants heart about cervical cancer and 8.5% heart about HPV. A large number (759) of respondents worried to develop cervical cancer and 53.7% thought they were at risk of developing cervical cancer. However only 69.05% didn't know that cervical cancer is preventable and 80.1% didn't know what is the contamination mode. Despite reporting family history of cervical cancer by 20 respondents, only 93 (11.07%) have been screened for cervical cancer. However, almost all of participants (96.67%) would accept to be screened for cervical cancer.

**Table 2:** Knowledge, attitude and practices of participants on cervical cancer

	Frequency	%
**Heart about cervical cancer**		
No	301	35.8
Yes	539	64.2
**Heart about HPV**		
No	769	91.5
Yes	71	08.5
**Is cervical cancer preventable**		
No	580	69.05
Yes	260	30.95
**Know contamination mode**		
No	673	80.1
Yes	167	19.9
**Know how to prevent cervical cancer**		
No	274	32.6
Yes	566	67.4
**Think at risk of cervical cancer**		
No	389	46.3
Yes	451	53.7
**Worry to develop cervical cancer**		
No	81	09.6
Yes	759	90.4
**Will accept to be screened**		
No	28	3.33
Yes	812	96.67
**Screened for cervical cancer**		
No	747	88.93
Yes	93	11.07

Table 3 shows that knowing contamination mode (OR = 7.76), family history of cervical cancer (OR = 4.59), having heart about cervical cancer (OR = 0.08), having heart about HPV (OR = 0.17), knowing that cervical cancer is preventable (OR = 4.47) were associated with screening history.

**Table 3:** Factors associated to cervical cancer screening attendance

Factors	Not Screened	Screened	OR 95%CI	p-value
**Age at first sex**				
≤19	562	71	0.94 (0.56-1.56)	0.41
≥20	185	22		
**Oral contraceptive use**				
Yes	320	63	0.35 (0.22-0.56)	0.000
No	427	30		
**Know contamination mode**				
No	634	39	7.76 (4.91-12.28)	0.000
Yes	113	54		
**History of STI**				
No	416	41	1.59 (1.00-2.52)	0.01
Yes	331	52		
**Family history of cervical cancer**				
No	734	86	4.59(1.78-11.83)	0.002
Yes	13	7		
**Heart about cervical cancer**				
No	296	5	11.22(4.77-26.39)	0.000
Yes	451	88		
**Heart about HPV**				
No	701	68	1.68(1.13-1.45)	0.000
Yes	46	25		
**Is cervical cancer preventable**				
No	545	35	4.47(2.85-7.00)	0.000
Yes	202	58		
**Think at risk of cervical cancer**				
No	355	34	1.57(1.00-2.47)	0.02
Yes	392	59		
**Worry to develop cervical cancer**				
No	69	12	0.68(0.35-1.32)	0.13
Yes	678	81		
**Will accept to be screened**				
Yes	720	92	0.28(0.03-2.15)	0.16
No	27	1		

Table 4 show that using multivariate analysis, think to be at risk of developing cervical cancer, heart about cervical cancer, know contamination mode, heart about HPV and use of oral contraceptive pills are significantly associated with screening history.

**Table 4:** Multivariate analysis of factors associated with cervical cancer screening

Factors	OR 95% CI	Coefficient	SE	Z Statistic	P-value
Think at risk of cervical cancer	1,03(0.62-1.70)	0.03	0.25	0.14	0.88
Heart about cervical cancer	5,70 (2,21-14,69)	1.74	0.48	3.60	0.00
Know contamination mode	3.81 (2.27-6.39)	1.33	0.26	5.07	0.00
Heart about HPV	2.05 (1.11-3.81)	0.72	0.31	2.29	0.02
Oral contraceptive use	2.06 (1.25-3.39)	0.72	0.25	2.84	0.00

## Discussion

This study has shown that cervical cancer screening rate is 11.07%. Our screening rate is higher than the national rate which was 5.5% in 2010 [5]. This might be explained by the fact that our study population is living in Ouagadougou where the screening service is available. But this rate is lower than in some African countries [18]. In develop countries such as USA, screening rate can reach more than 82.4% [19]. Similarly to many studies, we found that reason for not been screened are don't know where to go for screening, think that they are not at risk of developing cervical cancer, husband not allowed. Our study found that 35.8% of participants had never heard about cervical cancer and 91.5% had never heard about HPV. We also found that 69.05% did not know that cervical cancer is preventable, 80.1% did not know contamination mode of HPV and 67.4% did not know how to prevent cervical cancer. The knowledge of cervical cancer was low and screening attendance is associated with having knowledge such as knowing contamination mode, knowing that cervical cancer is preventable. A number of studies from other Sub-Saharan African countries have similarly found that women who lack awareness of cervical cancer are less likely to participate in screening service [18] and are thus at increased risk of developing cancer. We found that 89% of respondents have not been screened because they don't know where to go, they think they are not at risk and there are afraid of pain and husband don't allowed. Our findings are similar to other study which also found that reasons for not wanting to be screened are cannot have cervical cancer, husband is against it and screening expensive [20].

This lack of biomedical knowledge may partly be explained by the fact that cervical cancer, despite being the most common female cancer in Sub-Saharan Africa, is a rare condition that has not been prioritized by the national health system, advocacy programs have therefore not focused on cervical cancer. More diverse strategies should be employed to convey educational health messages which take into account the women's socio economic and cultural background. In relation it should be born in mind that experience from developed and developing countries have shown that conveying message via word of mouth and via audio visual channels are effective in making women more aware of cervical cancer and screening possibilities. In addition health education through trained lay persons in community centers should also be considered as this has been reported to be an effective method. All women should be encouraged to participate in a cervical screening programme at least once (between the age of 30 and 55 years). Public health education needs to be directed specially at the men folk to allow their wives to attend hospital to take care of their health need with or without their consent. It was found out in this study, parallel to the studies carried out, the rate of screening is higher among women whose family or relative have a story of cervix cancer and women thinking that they are in the risk of cervix cancer 46.3% think that they are not at risk of developing cervical cancer. It is important to correct their wrong perception about cervical cancer as all sexually active women are at risk.

## Conclusion

In conclusion, we determined that knowledgeable about cervical cancer and rate of women screened were very low. In addition to, having knowledge about cervical cancer, use of contraceptive method, history of cervical cancer in women's family or relatives, having STI, and thought herself in cervical cancer risky group were found to be related with their condition of being screened. According to the results of the research, it is needed to have screening programs suitable to country condition and to put forward cervix cancer prevalence. During this period, it is necessary to educate women, and to give them consultation service, and to suggest them go for screening test to improve their consciousness about screening with the aim of early diagnosis and prevention of cervix cancer. Advance studies with larger populations may provide detailed information in determining beliefs and attitudes concerning cervical cancer and having been screened. Special attention should be paid to lack of knowledge of cervical cancer also contributed in preventing women from attending cervical cancer screening. Women's perception and notions about cervical cancer need to be further assessed to develop communication strategies that take a broader cultural framework into account. Providing education and information orally as well as improving access to more.
